# COgnitive behavioural therapy vs standardised medical care for adults with Dissociative non-Epileptic Seizures (CODES): a multicentre randomised controlled trial protocol

**DOI:** 10.1186/s12883-015-0350-0

**Published:** 2015-06-27

**Authors:** Laura H. Goldstein, John D. C. Mellers, Sabine Landau, Jon Stone, Alan Carson, Nick Medford, Markus Reuber, Mark Richardson, Paul McCrone, Joanna Murray, Trudie Chalder

**Affiliations:** Department of Psychology, Institute of Psychiatry, Psychology and Neuroscience, King’s College London, De Crespigny Park, London, SE5 8AF UK; South London and Maudsley NHS Foundation Trust, Denmark Hill, London, SE5 8AZ UK; King’s College London, Institute of Psychiatry, Psychology and Neuroscience, Department of Biostatistics, De Crespigny Park, London, SE5 8AF UK; Department of Clinical Neurosciences, Western General Hospital, Edinburgh, EH4 2XU UK; Department of Rehabilitation Medicine and Department of Clinical Neurosciences, Royal Edinburgh Hospital, Morningside Terrace, Edinburgh EH10 5HF, and University of Edinburgh, Edinburgh, UK; Clinical Imaging Sciences Centre, Brighton and Sussex Medical School, Falmer, Brighton, BN1 9RR UK; Academic Neurology Unit, University of Sheffield, Royal Hallamshire Hospital, Glossop Road, Sheffield, S10 2JF UK; King’s College London, Institute of Psychiatry, Psychology and Neuroscience, Department of Basic and Clinical Neuroscience, De Crespigny Park, London, SE5 8AF UK; King’s College London, Institute of Psychiatry, Psychology and Neuroscience, Department of Health Service and Population Research, De Crespigny Park, London, SE5 8AF UK; King’s College London, Institute of Psychiatry, Psychology and Neuroscience, Department of Psychological Medicine, De Crespigny Park, London, SE5 8AF UK

**Keywords:** Dissociative seizures, Psychogenic non-epileptic seizures, Randomised controlled trial, Cognitive behavioural therapy, Clinical trial, Conversion disorder

## Abstract

**Background:**

The evidence base for the effectiveness of psychological interventions for patients with dissociative non-epileptic seizures (DS) is currently extremely limited, although data from two small pilot randomised controlled trials (RCTs), including from our group, suggest that Cognitive Behavioural Therapy (CBT) may be effective in reducing DS occurrence and may improve aspects of psychological status and psychosocial functioning.

**Methods/Design:**

The study is a multicentre, pragmatic parallel group RCT to evaluate the clinical and cost-effectiveness of specifically-tailored CBT plus standardised medical care (SMC) vs SMC alone in reducing DS frequency and improving psychological and health-related outcomes. In the initial screening phase, patients with DS will receive their diagnosis from a neurologist/epilepsy specialist. If patients are eligible and interested following the provision of study information and a booklet about DS, they will consent to provide demographic information and fortnightly data about their seizures, and agree to see a psychiatrist three months later. We aim to recruit ~500 patients to this screening stage. After a review three months later by a psychiatrist, those patients who have continued to have DS in the previous eight weeks and who meet further eligibility criteria will be told about the trial comparing CBT + SMC vs SMC alone. If they are interested in participating, they will be given a further booklet on DS and study information. A research worker will see them to obtain their informed consent to take part in the RCT. We aim to randomise 298 people (149 to each arm). In addition to a baseline assessment, data will be collected at 6 and 12 months post randomisation. Our primary outcome is monthly seizure frequency in the preceding month. Secondary outcomes include seizure severity, measures of seizure freedom and reduction, psychological distress and psychosocial functioning, quality of life, health service use, cost effectiveness and adverse events. We will include a nested qualitative study to evaluate participants’ views of the intervention and factors that acted as facilitators and barriers to participation.

**Discussion:**

This study will be the first adequately powered evaluation of CBT for this patient group and offers the potential to provide an evidence base for treating this patient group.

**Trial registration:**

Current Controlled Trials ISRCTN05681227

ClinicalTrials.gov NCT02325544

## Background

Dissociative seizures (DS) superficially resemble epileptic seizures or syncope but are not associated with ictal electroencephalographic (EEG) discharges. They are episodes of impaired self-control associated with a range of motor, sensory, and mental manifestations. They are one of the three common causes of Transient Loss of Consciousness [1]. Other names for these phenomena include ‘psychogenic non-epileptic seizures’ , ‘Non-Epileptic Attack Disorder’ (NEAD), ‘non-epileptic seizures’ , ‘functional seizures’and the more pejorative ‘pseudoseizures’ , to name but a few. Approximately 12-20 % of patients seen in epilepsy clinics may have DS [2] and such patients present a diagnostic and management challenge. Recent incidence estimates are 4.9/100,000/year [3]. Patients may previously have been misdiagnosed and treated for epilepsy; arrival at the correct diagnosis may take many years [4]. Long-term outcome (chronic disability and welfare dependence) has been noted to be poor in about 70 % of patients [5]. The vast majority of patients with DS are thought to have symptoms that are not deliberately generated. Therefore, they would receive diagnoses of somatoform disorder, conversion disorder, functional neurological symptom disorder or dissociative disorder under current classification systems [6–8].

DS are associated with high rates of psychiatric comorbidity (e.g. anxiety, depression, maladaptive personality traits and post-traumatic stress disorder) (e.g. [9]). Patients with DS are also vulnerable to other functional somatic symptoms such as chronic pain or other functional neurological symptoms [10] and have a slightly raised risk of non-seizure-related mortality [11]. They may undergo unnecessary, costly and potentially harmful tests and interventions and may sustain injuries during their seizures. Quality of life (QoL) is lower than in patients with epilepsy (e.g. [12]) and QoL correlates with depression and somatic symptoms. Patients’ lifestyles can be severely restricted through fear of having seizures and high levels of avoidance behaviour [13, 14]. Patients may be taking anti-epileptic drugs (AEDs) unnecessarily, with associated risks for women of childbearing age. A US study [15] evaluated the six-year cost pre-diagnosis to be in excess of $25,000/patient (approximately €23,587) and US lifetime costs were estimated at $110–920 m (approximately (€103.8–868 m). In Ireland, [16] the average annual cost per person with undiagnosed DS was calculated to be €5429.30 (i.e. approximately $5755), assuming that the average time taken to reach a diagnosis was five years. After correct diagnosis, a reduction in medical service use with attendant cost reductions may follow (e.g., [17, 18]).

Whilst psychotherapy is currently viewed as the treatment of choice [19], the evidence for its effectiveness is extremely limited [20–23]. The limited evidence to support the use of psychotherapy for patients with DS has come from a number of small uncontrolled studies and pilot RCTs [24, 25] which suggest the potential efficacy of Cognitive Behavioural Therapy (CBT). Our group’s manualised CBT treatment for DS has, in a pilot RCT [24], shown the potential to reduce DS frequency compared to standard medical care. In that study, 66 patients with DS were randomized to receive either CBT (plus standard medical care) or standard medical care alone. The primary outcome was seizure frequency at end of treatment and at 6-month follow-up. In an intention-to-treat analysis, seizure reduction following CBT was superior at treatment end (group x time interaction p <0.0001). At follow-up, there was a trend for the CBT group to be more likely to have experienced 3 months of seizure freedom (odds ratio 3.125, *p* = 0.086). Both treatments led to some improvement in psychosocial functioning.

The lack of a clear evidence base is reflected in the extremely variable care provision for DS patients in the UK, with currently no rational basis on which to decide whether psychotherapy and which type should be recommended for this patient group. A survey of UK healthcare professionals working with DS patients reflected this variability in that only one third of respondents indicated that they could refer all their patients for psychotherapy, while clinicians’ knowledge of what type (s) of psychotherapy might be available and where was also mixed [19]. One third of respondents indicated that fewer than half their patients would be offered ≥1 psychotherapy session. Despite an increasing acknowledgement of the interface between neurology and psychiatry, neuropsychiatry care pathways are relatively under-developed [26]. Indeed, there is variable involvement of psychiatrists and psychologists in the assessment and management of DS patients. This is despite increased recognition that neuropsychiatric disorders, such as DS, may cause distress to patients and their carers, disability, burden and loss of productivity [26]. More evidence would contribute to national and local discussions about what treatments should be provided for patients with DS patients.

Our study follows MRC Guidelines for complex interventions [27]. We have already completed a proof of principle RCT and obtained preliminary evidence of efficacy [24]. Thus, the next step is to evaluate the clinical and cost effectiveness of our intervention and assess its generalisability in an adequately powered, pragmatic, multi-centre RCT.

### Aims and objectives

The overall aim of the CODES Trial is to evaluate the clinical and cost effectiveness of specifically adapted CBT (plus Standardised Medical Care-SMC) in comparison to SMC alone for outpatients with DS, within a pragmatic, multi-centre RCT.

Our primary objective is to evaluate the effectiveness of CBT (plus SMC) compared to SMC alone in reducing DS frequency (our primary outcome) at 12 months post randomisation.

Our secondary objectives are to evaluate:

1. the effectiveness of CBT plus SMC compared to SMC alone in reducing subjective DS severity and disability and promoting seizure freedom, health-related quality of life, and psychosocial and psychological well-being at 12 months post randomisation;
2. the effectiveness of CBT plus SMC compared to SMC alone in reducing health service use at 12 months post randomisation;
3. the cost-effectiveness of CBT plus SMC compared to SMC alone at 12 months post randomisation;
4. patients’ global change as a result of treatment (Clinical Global Impression (CGI) [28] change score);
5. their satisfaction with treatment;
6. DS patients’ subjective experiences of CBT vs. SMC, determined from qualitative interviews;
7. the treatment fidelity of our manualised DS-specific CBT treatment across different therapists and its implications for rollout in the NHS.

## Methods and design

### Trial design

The study is a multicentre, pragmatic, parallel group randomised controlled trial (RCT) of CBT plus SMC vs SMC alone. After an extended screening phase to identify eligible patients, those patients found to fulfil eligibility criteria and who consent to the RCT are randomised at a 1:1 ratio to either treatment arm. Patients will be assessed at baseline (pre-randomisation) and six months  post randomisation, with final outcome assessed at 12 months post randomisation.

The study will take place in neurology services and neuropsychiatry/mental health services in England, Scotland and Wales. The current clinical neurology services from which we will recruit patients for the screening phase are those secondary and tertiary clinical services seeing patients presenting with seizures that require diagnostic assessment and review; the clinical psychiatry services from which we will subsequently consent patients for the RCT are those that have interest and experience in treating patients with DS. To date we have approval to recruit participants from 25 neurology and 15 psychiatry services.

### Target population

**Fig. 1:**
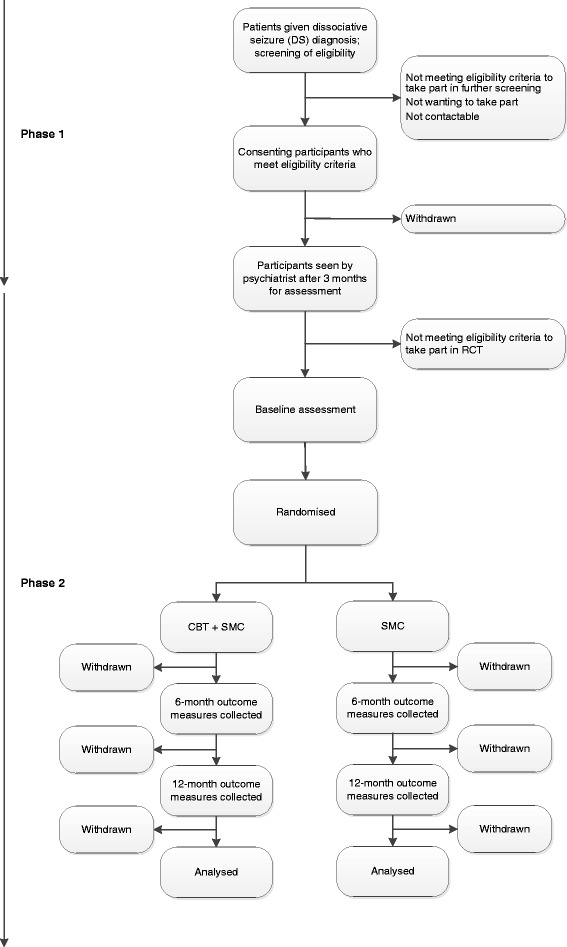
Study flowchart: phase 1 = initial screening phase following diagnosis in neurology/specialist epilepsy services of identified patients; phase 2 = further screening following psychiatric assessment, baseline assessment, randomisation, treatment and follow-up; CBT = Cognitive Behavioural Therapy; SMC = Standardised medical care

The target population for this pragmatic trial is adult outpatients with DS which persist following diagnosis by neurologists/epilepsy specialists. Since our study comprises more than one stage to ascertain eligibility, we describe inclusion and exclusion criteria for each stage below. A summary of the participant flow through the study is shown in Fig. 1.

### Recruitment

In order to randomise 298 patients (see justification below) we need to identify a substantially larger pool of DS patients in neurology clinics, since in addition to other potential exclusion criteria, approximately 14 % of DS patients will be seizure free three months post diagnosis [29]. In order to allow for ineligibility of patients attending the psychiatry appointments, we envisage needing to recruit 501 patients who meet eligibility in the screening phase of the study.

### Screening phase

Adults with DS will receive their diagnosis and an information leaflet on DS from a neurologist/epilepsy specialist.

Participants will be included in the in the screening phase if they:

- are adults (≥18 years) with DS that have continued to occur within the previous 8 weeks and have been confirmed by video EEG telemetry or, where not achievable, clinical consensus; patients who have chronic DS can be included if they have been seen by the relevant Study Neurologist who has reviewed their diagnosis and communicated this to them according to the Study protocol;
- have no documented history of intellectual disabilities;
- are able to complete seizure diaries and questionnaires;
- are willing to complete seizure diaries regularly and undergo psychiatric assessment 3 months after DS diagnosis;
- are able to give written informed consent.

Participants will be excluded from the screening phase if they:

- have a diagnosis of current epileptic seizures as well as DS (where current is defined as an epileptic seizure within the previous year). Patients with both DS and ES have been included in small studies (e.g. [30, 31]) but there is no method for verifying that patients can accurately differentiate between epileptic seizures and DS;
- are unable to keep seizure records or complete questionnaires independently;
- meet DSM-IV [6] criteria for current drug/alcohol dependence;
- have insufficient command of English to later undergo CBT or complete questionnaires without an interpreter. Reasons for this include the need to self-rate secondary outcomes using scales not validated for non-English speaking populations, the considerable cost and uncertainty of being able reliably to engage sufficiently competent interpreters, and the need to demonstrate the delivery of therapy in terms of competence of therapists and adherence to the therapy manual;
- are currently having CBT for another disorder, if this will not have ended by the time that the psychiatric assessment takes place;
- have previously undergone a CBT-based treatment for dissociative seizures at a trial participating centre.

If patients are deemed eligible for the screening stage of the study they will be given study information and, if interested, will be contacted by phone and/or letter by a research worker. Following further eligibility checks against the above criteria and explanation of the study they will then be consented to the screening phase of the study. In addition to the provision of demographic data, participants’ DS seizure occurrence (in terms of self-reported frequency and severity) will be monitored fortnightly using seizure diary completion. Data will be collected by a research worker by phone, email, text or post, depending on the participant’s preference.

Three months after receiving their diagnosis of DS, those meeting eligibility criteria so far will be reviewed by a liaison psychiatrist or neuropsychiatrist with an interest and expertise in DS, to carry out the final screen for eligibility for the RCT.

Participants will be included in the in the RCT if at the psychiatrist assessment visit and pre-randomisation:

- they are adults (≥18 years, no upper age limit) with DS, initially recruited at point of diagnosis;
- they are willing to continue to complete seizure diaries and questionnaires;
- they have provided regular seizure frequency data following receipt of DS diagnosis;
- they are willing to attend weekly/fortnightly sessions if randomised to CBT;
- they and the clinician consider that randomisation is acceptable in the participant’s case;
- they are able to give written informed consent.

Participants will be excluded from the RCT if, at the psychiatric assessment visit and pre-randomisation, they:

- are having current epileptic seizures as well as DS, for reasons given above;
- have not had any DS in the 8 weeks prior to the psychiatric assessment;
- have previously undergone a CBT-based treatment for dissociative seizures at a trial participating centre;
- are currently having CBT for another disorder;
- have active psychosis;
- meet DSM-IV [6] criteria for current drug/alcohol dependence; this may exacerbate symptoms/alter psychiatric state and health service use and affect recording of seizures;
- have current benzodiazepine use exceeding the equivalent of 10 mg diazepam/day;
- are thought to be at imminent risk of self-harm, after (neuro) psychiatric assessment or structured psychiatric assessment by the Research Worker with the MINI, followed by consultation with the psychiatrist.
- have a known diagnosis of Factitious Disorder.

The psychiatrist will go through the diagnosis again, undertake a clinical assessment and provide them with a further, more extensive information booklet on DS. Those patients who have continued to experience DS in the previous eight weeks and who meet the further study criteria listed above will, with their agreement be contacted by a research worker and then, if willing, consent to take part in the trial, undergo a baseline assessment and then be randomised to receive either 12 sessions of CBT (plus a booster session) plus SMC or SMC alone.

### Interventions

#### Standardised medical care (SMC)

While the provision of standard medical care to DS patients in the UK is variable [19], different specialities contribute to standard care in specific ways. Through the development of a protocol and the development of new as well as use of existing materials, we have established key approaches to the delivery of what is best considered here as Standardised Medical Care (SMC). This will contain elements documented elsewhere and shown to be achievable and acceptable to patients [29]. This approach, involving the provision of a prompt sheet, detailed leaflet for clinicians and briefing sessions for medical staff has been shown to work in other studies [29]. The key elements of SMC are described below.

SMC will be provided to study patients by neurologists and psychiatrists. Neurologists will generally undertake the assessment and investigation and then make and deliver the diagnosis, which is the first step in the treatment pathway [22]. Psychiatrists will provide further assessment of aetiological and maintenance factors, assess and treat complex co-morbidity and give routine advice on seizure management and adaptation but without using CBT techniques. As part of SMC we have devised two information booklets about DS to be given to patients, to supplement the information given to them during their clinical appointments by the neurologists and psychiatrists. These booklets have been created with contributions from service users and a Hospital Information Officer to increase ease of reading and are as follows:

1. Dissociative Seizures Factsheet (Neurology) is a short booklet for neurologists to give to patients when they deliver the diagnosis.
2. Dissociative Seizures Factsheet (Psychiatry) is more detailed written information to supplement SMC psychiatric assessment and treatment. The aim is for this to be given to the patient at the time of the initial psychiatric assessment.

### Neurologists’ delivery of SMC

Neurologists will be expected to assess the patient in their usual way to determine the nature of the patient’s seizures. In some cases it will be possible to make a secure diagnosis on the basis of the history, witness history and physical assessment. In other cases mobile phone footage, EEG or video EEG may be required to make the diagnosis. Where available and practical an EEG with concurrent video is the most reliable way to make the diagnosis but it is not mandatory. Likewise, neuroimaging is not mandatory and it is anticipated that this will be carried out only according to clinical need. Where video EEG telemetry is not available, we will accept a consensus diagnosis that either involves the agreement between two neurologists in the clinical service dealing with the patient or between the study neurologist and one neurologist within the project team.

Neurologists will not be expected to carry out a standardised psychiatric assessment. However, as with all patients attending a neurological service, if there are clearly recognisable psychiatric risks related to self-harm, harm of others or psychosis the neurologist will refer to the relevant psychiatric services, or ask the patient’s general practitioner (GP) to do so.

All neurologists will be expected to give patients a copy of an information booklet about Dissociative Seizures (see above). In addition to information about DS this will include direction to self-help information (e.g. www.nonepilepticattacks.info, www.neurosymptoms.org).

The neurologist will be expected to provide the following information:

1. An explanation of what DS are: i) i.e. provide the patient with a diagnostic label; ii) explain that the patients do not have epilepsy and why, drawing particular attention to positive aspects of the diagnosis (e.g. that this is not epilepsy) and explaining why tests are confirmatory of the diagnosis; iii) explain that the person’s attacks are genuine and they are not suspected of “putting on” or ‘imagining’ the attacks; iv) explain that the disorder is common; v) explain that the condition is potentially reversible.
2. An explanation of the mechanism underlying DS: We allow for the possibility that neurologists may wish to talk about the mechanism of the attacks being a ‘trance-like’ state called dissociation, similar to that seen in hypnosis. Individual explanations will vary according to the patient’s presentation. We advise the neurologist against using any explanation that leaves the patient thinking that the doctor does not believe them or thinks they are just ‘making it up’.
3. An explanation for referral to a psychiatrist: We recommend that the neurologist emphasises the following issues to the patient in discussing the referral to a psychiatrist and encourages them to attend: i) the doctor may be a psychiatrist but they will not think the patient is ‘crazy’; ii) the psychiatrist knows about DS and has successfully helped other patients with the problem; iii) psychosocial factors are often important in understanding DS and part of the reason for referral is to explore this further; iv) to assess factors that might be maintaining the attacks.

There are other aspects of the initial neurological consultation that may vary according to the patient but could involve: i) explaining that antiepileptic drugs do not help DS, can have serious long-term side effects and should be withdrawn gradually; ii) explaining that talking treatments may be helpful for some people but the evidence is currently uncertain as to whether it is worthwhile; iii) providing explanations to family and friends about the diagnosis, and what to do when the patient has an attack; iv) providing general information about distraction techniques; and v) discussing driving regulations.

### Further neurology follow-up

We are recommending that the neurologists offer at least one further neurology follow-up visit (although fewer or more are allowable) which may typically cover the following topics: i) overall general review of progress; ii) checking the patient’s understanding of the diagnosis and explaining it again if necessary; iii) supervision of AED withdrawal; iv) management of any comorbid physical conditions; reassessment of major psychiatric risk such as self-harm or psychosis; v) recommendations for antidepressant or anti-anxiety medication prior to the first visit with the psychiatrist if clinically indicated; and vi) completion of forms about driving or from the Department of Work and Pensions if requested by those agencies.

#### Psychiatrists’ delivery of SMC

Psychiatrists’ provision of SMC of patients begins after diagnosis with an outpatient appointment as close as possible to three months after the neurological assessment.

The initial clinical psychiatric assessment will include the following components. It should include: i) a reiteration of all of the points covered by the neurologist at diagnosis, including checking the patient has received the information booklet about Dissociative Seizures that was delivered by the Neurologist and direction to self-help information; ii) provision of a more detailed booklet “Dissociative Seizures Factsheet (Psychiatry); iii) acknowledgement of fears about a psychiatric label; iv) clinical assessment of relevant axis 1 (e.g. depression, anxiety) and axis 2 (personality disorder traits) psychiatric disorders, including an assessment of the risk of self-harm/suicide; v) explanation and treatment of any psychiatric comorbidity which may include provision of psychopharmacological treatment (e.g., antidepressants) or general treatment as required; vi) explanation of any other functional somatic symptoms, general advice about management and referral to physiotherapy if appropriate for mobility problems; vii) discussion of factors emerging from the clinical history that seem to have aetiological significance: relevance of predisposing, precipitating and perpetuating factors in their case if apparent; viii) provision of general information about any warning symptoms and distraction but specific techniques will not be discussed so that this does not become therapy; and ix) liaison with other mental health professionals involved in the patient’s case as appropriate but referrals for other psychotherapeutic input (including use of CBT techniques) specifically for DS will not be made. The emphasis should be on psycho-education and management of comorbid psychiatric conditions in the normal way. The session may also include involvement of family or friends in the above steps as required; encouragement in social activities; return to college/work as appropriate with any necessary liaison with work/school/college to explain the disorder and assist with the correct management of DS in these environments if appropriate; completion of forms about driving or the Department of Work and Pensions if requested by those agencies.

Further SMC by psychiatrists will include support, consideration of psychiatric comorbidities and any associated drug treatment and general review but CBT techniques for DS will not be discussed.

Neurologists and psychiatrists will be provided with a prompt sheet containing the essential information to be covered during sessions, an expanded description of information they might provide to patients, a set of Frequently Asked Questions and access to the secure section of the study website (www.codestrial.org). On the website they can access trial materials and videos demonstrating delivery of the diagnosis by neurologists and explaining randomisation.

Despite some local variation (due to factors such as commissioning differences and clinicians’ preferences), following the initial neurology assessment and the psychiatric assessment we anticipate up to two neurology SMC sessions and three-to-four psychiatry SMC sessions. However, due to the pragmatic nature of the study we are not prescribing the number of sessions.

#### Cognitive Behavioural Therapy (CBT)

CBT will be delivered over 12 sessions (each approximately one hour in length) over a 4–5 month period, with one booster session at 9 months post randomisation.

Our model has been developed from a single case study [32], trialled in an open label study [33] and then in a pilot RCT [24]. Thus, based on our pilot RCT [24] we will assess a 12-session (plus one booster session) package of CBT specifically modified for treating DS. This has been described [34] and tested by our group [24, 33].

The model is based on Lang’s [35] two-process fear escape-avoidance model and conceptualises DS as dissociative responses to cues (cognitive/emotional/physiological or environmental) that may (but not in all cases) have been associated in some cases with distressing or life-threatening experiences, such as abuse or trauma at an earlier stage in the person’s life, or following events such as panic attacks or syncope and which have previously produced intolerable feelings of fear and/or distress [34]. There are essentially five stages to the treatment; engagement and rationale giving; teaching and use of seizure control techniques; reducing avoidance using exposure techniques; dealing with seizure-related cognitions, facilitating the processing of emotions and where appropriate trauma; and relapse prevention.

Thus, treatment includes helping the patient to: i) develop a more coherent understanding of their DS; ii) develop an understanding of the interrelationship between cognitive, emotional, physiological and behavioural aspects of their DS; iii) understand factors maintaining the occurrence of their DS; iv) learn how to interrupt the behavioural, cognitive or physiological responses occurring prior to or at the start of the seizures; v) engage in previously avoided activities, address negative thoughts and illness attributions maintaining seizures; vi) deal with previous traumatic experiences, anxiety, low mood or low self-esteem if present, and vii) become more independent and understand the role of significant others in their illness.

Sessions include typical CBT techniques and here, importantly, completion of seizure diaries. Although our treatment manual outlines the content of the 12 sessions in detail and provides hand-outs to be given to patients that supplement the content of the therapy sessions, the structure allows the treatment to be formulation-based so that particular issues raised in therapy that might be maintaining seizure occurrence (e.g. trauma-related issues) can be addressed.

Sessions will be delivered at participating National Health Service (NHS) sites. Attendance at CBT sessions will be monitored, as will reasons for rescheduling the sessions or for non-attendance. We will also record the occurrence of DS during sessions that lead to disruption of the session or injury. Therapists will rate patients’ completion of homework tasks and adherence to the therapeutic model, on a sessional basis. If DS cease early in treatment we will encourage continued attendance by patients at sessions to address other significant aspects of their presentation and to focus on relapse prevention.

The CBT will be delivered by CBT therapists (health professionals, i.e. clinical psychologists/nurse therapists or other professions allied to medicine-already trained in CBT). They will be trained to deliver CBT for DS. Before treating any trial patients, therapists will attend a three-day workshop, specifically focusing on DS. The workshops will include DS-specific knowledge and skills. Therapists will receive a combination of group supervision, four-to-six weekly and, where required, individual supervision. With participants’ consent, all CBT sessions will be audio-recorded. Some recordings will be used by supervisors to provide feedback to therapists to ensure adherence to the treatment model and specific treatment approach. Any significant deviations from the manual will be noted and fed back to the therapist. By adopting such an approach our experience from other illnesses suggests we can achieve expected treatment outcomes and have very little therapist effect [36]. We will also use the audio recordings to rate therapist competence, therapeutic alliance and adherence to the manualised therapy.

### Outcome measures

Our primary evaluation of treatment is at 12 months post-randomisation. However, we will also collect outcome measures at 6 months post randomisation and include these in our analyses.

Our primary outcome measure is monthly DS frequency operationalised as seizure occurrence over the previous four weeks. This is a discrete variable that comprises a count of seizures and therefore will reflect all participants’ outcomes, whether they improve or not during the study. Seizure frequency has been used as an outcome measure in other studies of psychological interventions for DS (e.g. [24, 25, 30, 37, 38]). This will be recorded by patients in seizure diaries, as has been done in other studies of psychotherapy for DS. We will collect seizure frequency data from the patients every two weeks by whichever means they find acceptable (diaries, text/phone/online). We will also request an overall self-report estimate of DS frequency in the previous four weeks from participants at baseline and the two follow-up time points, to allow for missing diary data.

Our study seeks to evaluate the effectiveness and cost-effectiveness of CBT+ SMC vs SMC alone in a number of domains. Thus we have selected a range of clinical and economic secondary outcome measures. These are listed in Table 1. In view, however, of the likelihood of psychiatric comorbidities moderating outcome in this patient group, we also include at baseline a screening measure of personality disorder, the Standardised Assessment of Personality Abbreviated Scale, Self Report version (SAPAS-SR) [39, 40] and a structured psychiatric screening instrument (the Mini International Neuropsychiatric Interview; MINI v6.0 [41]); the prerandomisation assessment will also include a single item measure of treatment preference and a measure of expectations of treatment outcome, both of which may also moderate outcome.

**Table 1:** Primary and secondary outcome measures and times of data collection

Outcome variable/domain	Standardised measure/other	Putative moderators or mediators of treatment effects on clinical outcome	Clinical/economic outcomes	TO	T1	T2
PRIMARY OUTCOME						
Seizure frequency	Seizure diary and self-report		X	X	X	X
SECONDARY OUTCOMES						
*Seizures*	Seizure severity; Two items measuring subjective severity (intensity) and bothersomeness from the Seizure Severity Scale [53]		X	X	X	X
	Longest period of seizure freedom between T1 & T2; seizure diary and self-report		X			X
	Seizure freedom for last 3 months of study; seizure diary and self-report		X			X
	>50 % reduction in seizure frequency; seizure diary and self-report		X		X	X
	Informant’s rating of patient’s seizures; Rating as to whether participant’s seizures are better/same/ worse or whether they are seizure free		X		X	X
*Health-related Quality of Life (QoL)*	SF-12v2 (12-item measure of health-related QoL) [50]		X	X	X	X
*Psychosocial functioning*	Work and Social Adjustment Scale (5-item scale to measure patients’ own perceptions of the impact of DS on their functioning in terms of work, home management, social leisure and private leisure activities, family and other relationships) [54]		X	X	X	X
	Avoidance of people, places and situations (3-item locally devised scale)	Mediator		X	X	X
*Psychiatric symptoms and psychological distress*	GAD7 (7-item scale to measure anxiety) [55]		X	X	X	X
	PHQ9 (9-item scale to measure depression) [56]		X	X	X	X
	Modified PHQ15 (measure of other somatic symptoms) [57, 58]		X	X	X	X
	CORE-10 (10-item scale; general measure of psychological distress including risk) [59]		X	X	X	X
	12-item Beliefs About Emotions Scale [42]	Mediator		X	X	X
*Clinical impression of improvement*	Clinical Global Impression (CGI) [28] Change score		X		X	X
	Clinical Global Impression (CGI) [28] Change score rated by clinician (psychiatrist or neurologist)		X			X
	Clinical Global Impression (CGI) [28] Change score rated by CBT therapist at end of session 12 of CBT		X			
*Satisfaction with treatment and beliefs relating to diagnosis and treatment*	Single item measure of satisfaction with treatment		X		X	X
	Belief in diagnosis of DS (single item; 11 point scale)	Mediator and/or moderator		X	X	X
	Belief in having been given the correct treatment (single item; 11 point scale)	Mediator		X	X	X
*Health economics*	Client Service Receipt Inventory [46]: Formal and informal health service use		X	X	X	X
	EQ-5D-5L (5-item, 5 level measure) [49]		X	X	X	X
	Linkage data sets^a^ from Health and Social Care Information Centre (Hospital Episode Statistics) eDRIS (NHS National Services Scotland Information Services Division (ISD) and NHS Wales Informatics Service		X	X	X	X

T0 = Baseline (prerandomisation) (face to face); T1 = 6 month follow-up (postal); T2 = 12 month follow-up (face-to face)

^a^All data sought electronically

### Other baseline data

We will describe the target population in a number of ways. Demographic information (including age, gender, relationship status, presence/absence of dependants and/or a carer, ethnicity, postcode to evaluate Indices of Multiple Deprivation, employment status, receipt of state benefits, previous medical help sought for a mental health problem, previous diagnosis of epilepsy and current receipt of anti-epileptic drugs) will be collected at the beginning of the screening phase. This will be supplemented with information on self-reported pre-existing medical conditions at the pre-randomisation assessment so that reports of any newly-diagnosed reported medical conditions or symptoms following randomisation can be compared to these in order to identify adverse events. Information on potential adverse events will be collected systematically at 6 and 12 months post randomisation by the research workers. We will also record demographic data and the qualifications and clinical experience of the neurologists, psychiatrists and therapists in the study.

As indicated in Table 1, we will investigate potential mediators of the treatment effect by evaluating responses at baseline and the 6 and 12 month follow-up time points on the Beliefs About Emotions Scale [42], three locally-developed questions to measure avoidance of people, places and activities due to DS, and a single item scale for participants to measure their confidence in the treatment they have received; we will investigate whether a further item rating their confidence in their diagnosis of DS may act as a moderator and/or mediator of the treatment effect.

### Sample size calculation

We based our power calculation on the effect size obtained in our pilot RCT study [24]. This data represented the largest study of this kind to date and importantly included a control group. Our previous pilot trial’s analysis, which controlled for pre-randomisation seizure frequency, reported a standardised effect size for the reduction in seizure frequency under CBT compared to SMC at the end of CBT treatment of Cohen’s d = 0.75 (log scale). Our previous study also detected a more conservative and moderate effect size of Cohen’s d = 0.42 on the log-scale at a follow-up time more directly comparable to the current 12-month post randomisation point (median seizure frequency in the CBT group: 12 at pre-randomisation, 1.5 at follow-up; median frequency in the SMC group: 8 at pre-randomisation, 5 at follow-up). We consider this effect to be clinically important and therefore base our power calculation on this moderate effect size. To detect an effect of d = 0.42 with 90 % power using a 2-sided *t*-test for log-frequencies at the 5 % significance level, we need 121 participants/group. The sample size must be inflated to allow for potential therapist effects within the CBT group, since each therapist will most likely treat several patients. Based on a typical therapist intraclass correlation coefficient of 0.02 [43] and 15 therapists delivering CBT (average workload 10 patients/therapist), 149 participants are needed per arm to achieve 92.6 % power. We will record pre-randomisation seizure frequencies and include this information as a covariate in the analysis model. This will increase the precision of our future intervention effect estimate. To account for this precision gain and the subsequent reduction in sample size requirement, we can apply a deflation factor to the estimated sample size [44]. We calculated the size of this deflation factor as 0.8367 based on a correlation of r = 0.42 between pre-randomisation and follow-up in DS frequencies [24]. Finally, in our pilot RCT, 7/66 patients were entirely lost at follow-up. We therefore need to inflate the sample size allowing for a more conservative rate of 17 % attrition at 12-month follow-up. Our final randomisation target is 298 participants (149 per arm).

### Randomisation and concealment

Randomisation will be carried out by the King’s Clinical Trials Unit (KCTU: www.ctu.co.uk) based at the Institute of Psychiatry, Psychology and Neuroscience, using a web-based system. Randomisation takes place once participants have consented to take part in the trial and baseline assessments have been undertaken. The unit of randomisation will be the individual participant. Stratified randomisation with randomly varying block sizes will be used to ensure 1:1 allocation within each of the locations of the neuro/liaison psychiatry clinics from which DS patients are recruited. Allocation will be concealed from the research workers who undertake trial data collection, and also from the trial statistician. The Trial Manager will receive notification of the allocation so that therapists can be informed that they need to arrange to see those participants requiring CBT. We will require research workers to indicate if they have become unblinded and when this occurred, so that outcome assessments can be undertaken by blinded assessors.

### Statistical analysis

Statistical analyses of the primary and secondary outcomes will be conducted after the database has been locked, with no interim analyses. All analyses will adopt the intention to treat principle. Descriptive statistics will describe the characteristics of the initial pool of patients in the screening phase, and also those entering the RCT. For the primary seizure frequency outcome, treatment effectiveness will be assessed by estimating the incidence rate ratio (IRR), comparing the CBT + SMC and SMC arms at the 12-month follow-up time point. Generalised linear mixed modelling (GLMM) assuming a Poisson distribution, a log-link and allowing for possible overdispersion will provide this estimate. The dependent variable of this analysis model will be seizure frequency at 6- and 12 months post randomisation. The explanatory variables will be baseline seizure frequency, randomisation stratifier (liaison/neuropsychiatry clinics), and trial arm, time (6 or 12 months post-randomisation) and trial arm x time interaction. All the available post-randomisation data is modelled simultaneously to gain precision. The model includes the interaction term to allow for time-varying treatment effects. The model also contains participant-varying random intercepts to account for the correlation between the two repeated measures, and will consider including random effects to account for effects of the doctor delivering SMC and therapist-varying intercepts in the CBT arm to account for therapist effects. The models are estimated using maximum likelihood analysis and will allow for missing outcome data under the missing at random (MAR) assumption. The effect of departures from this assumption on results will be assessed using sensitivity analyses [45]. Analyses of secondary outcomes will use a similar approach (for example, continuous outcomes such as quality of life will be analysed using a linear mixed model).

### Cost effectiveness analysis

We will undertake a cost-effectiveness analysis from a (i) health and social care and (ii) societal perspective (including lost productivity and informal care). The number and duration of CBT sessions will be centrally recorded and other service utilisation will be recorded with the Client Service Receipt Inventory [46] questionnaire at baseline, and at 6-and 12-month follow-ups. This will include primary and secondary care contacts, social care use, care from family members and medication. We will also record lost work time. Information from Hospital Episode Statistics will also be used to estimate hospital use.

Intervention costs for the CBT sessions will be based on factors such as salaries, overheads, training and supervision. Unit costs for other services will be obtained from national sources [47, 48]. Costs of lost work and informal care will be based on average wage rates but with alternative values used in sensitivity analyses. Costs will be combined with the primary outcome measure (change in DS frequency) and also QALYs generated from the EQ-5D-5L [49] using area-under-the-curve methods. In sensitivity analyses we will use the SF-6D, generated from the SF-12v2 [50], to derive QALYs via an algorithm developed by economists at the University of Sheffield [51]. If the intervention is less expensive and more effective than SMC then it will be ‘dominant’. If it is more expensive and more effective, incremental cost-effectiveness ratios will be constructed to show the extra cost incurred to achieve a one-unit reduction in DS frequency or one extra QALY. Uncertainty around cost-effectiveness estimates will be explored using cost-effectiveness planes (derived from incremental cost-outcome pairs from 1000 bootstrapped resamples) and cost-effectiveness acceptability curves (CEACs - derived using the net benefit approach). In addition to the use of the SF-6D, sensitivity analyses will also be conducted around the costs of the intervention, informal care and lost employment. There has been limited previous research in this area and this trial will provide evidence on the impact of CBT in DS patients over a one-year follow-up.

### Qualitative study

We will undertake a nested qualitative study to investigate the illness attributions, treatment preferences and experiences of trial participants. In-depth interviews with 20 patients selected across sites who have received CBT + SMC and 10 who have only received SMC will be undertaken to understand what they felt to be beneficial in terms of the interventions, and  what made it easy or more difficult for them to take part. We will also enquire about the extent to which they have been able to implement the content of the CBT, and their views of the content of the therapy. We will include participants who did not attend all the CBT sessions, as well as those who did. The interviews will take place in participants’ homes or will be office-based. The interviews will be digitally recorded and transcribed. Thematic Framework Analysis [52] will be carried out by at least two Research Workers under the supervision of an experienced qualitative researcher and rigour will be increased by them undertaking independent coding, followed by discussion meetings to agree a coding framework, to reduce bias in the interpretation of themes. Triangulation of the findings from the qualitative analysis with the results of the quantitative outcome measures will increase understanding of the trial process and may assist in understanding anomalies in outcomes.

### Data handling and monitoring

We will collect data on paper source data worksheets. Data will then be entered onto the InferMed MACRO online data entry system, on a study specific database designed and hosted at the KCTU. The system is compliant with Good Clinical Practice and FDA 21 CFR Part 11. Randomisation and post-randomisation information will be accessed directly by the trial statistician using CTU systems.

In addition to a Trial Management Group (TMG), monitoring of the trial activities and recruitment will be undertaken by the Trial Steering Committee (TSC) and the DMEC. The DMEC will monitor recruitment into both the initial screening (phase 1) and randomisation (phase 2) stages of the trial on a monthly basis. Monitoring by the study team, the Trial Manager and the KCTU will ensure that the trial complies with Good Clinical Practice and maintains scientific integrity. The Trial Manager will monitor data collection procedures and undertake source verification checking against the paper data records at regular intervals.

We have developed a protocol for monitoring adverse events during both the screening and RCT phases of the study. While we may become aware of adverse events at any stage of the study, research workers will specifically enquire about these at the 6-and 12-month post randomisation follow-up points. We anticipate a high rate of these events, given the likely psychiatric comorbidity in this patient group, but we will distinguish between serious adverse events that are likely/unlikely to be due to the intervention in the RCT phase of the study. At the end of the study, three independent scrutineers, with clinical experience of working with DS patients, will assess whether events were serious or non-serious.

## Discussion

To our knowledge this is the first adequately powered randomised controlled trial of a psychological intervention to treat DS, anywhere in the world. The study has considerable challenges given the different care services involved and the likely psychiatric comorbidity of patients, which may make study compliance difficult, although the collaboration of a large number of clinicians and researchers with expertise in DS may facilitate means to encourage compliance. The study is set in the context of the UK NHS and if it yields a positive outcome in terms of clinical and cost effectiveness, it will provide the opportunity for the NHS to commission evidence-based care for this group of patients, for whom the availability of care provision is currently notably inconsistent.

### Ethics approval

NRES Committee London-Camberwell St Giles. Reference number 13/LO/1595.

### Trial status

Ongoing (recruiting participants).

## Abbreviations

CBT: Cognitive behavioural therapy
DS: Dissociative seizures
SMC: Standardised medical care
